# Insights from ESMO Asia Congress 2024

**DOI:** 10.1016/j.lansea.2025.100554

**Published:** 2025-02-21

**Authors:** Thittayil Suresh Apoorv

Every year, the European Society for Medical Oncology (ESMO) holds an annual Congress focused on developments in the field of prevention treatment and care in cancer in Asia. This event provides an opportunity for health professionals based in Asia and other continents—working in any aspect of oncology—to learn and collaborate. As in previous years, the ESMO Asia Congress was held in Singapore from December 6-8, 2024. Compared to 2022, there were more Asian participants and increased engagement, highlighting the Congress's growing popularity and influence.

## Challenges in Asia

In the session “Prevention, early diagnosis, and interception programmes across the globe”, **Dr Eileen Poon (Singapore)** talked about the role of charity organisations in supporting breast cancer screening centres, which helps in making mammography affordable and accessible for people with low-income. **Dr Dilyara Kaidarova (Kazakhstan)** talked about Kazakhstan's efforts to cervical cancer cases. HPV vaccinations have increased and covered 85,000 girls as of now. Endoscopies are being conducted at an early stage and PAP smear is included in the screening, which has led to increased detection of cervical cancer cases and number of deaths is currently stable. Political will is the main reason for the change, with respect to more budget allotment for the screening, more polyclinics, and most importantly, politicians are keen to regularly know the status of cancer cases in Kazakhstan. **Dr Senthil Rajappa (India)** critically analysed the NIAGARA trial from an Asian perspective (durvalumab + gemcitabine + cisplatin [neoadjuvant] followed by durvalumab alone [adjuvant] for muscle-invasive bladder cancer). Dr Rajappa commented that overall survival benefit supported NIAGARA over CheckMate 274 trial (adjuvant nivolumab vs placebo). Also, although NIAGARA trial is a small step towards cure, it is a huge step for the scientific community. Unfortunately, access to durvalumab is a challenge in several Asian countries. Dr Rajappa concluded his talk with: “*NIAGARA would fall on some countries, but in other countries, it would trickle*” emphasising the importance of increasing accessibility of cancer drugs.

## Clinicians’ challenges

**Dr. Shona Nag (India)** highlighted the challenges in delegating genetic counselling tasks to highly skilled nurses in some hospitals, where language barriers related to English can complicate communication. Some oncologists presenting scientific posters at the Congress mentioned that the scarcity of research support and funding from governments posed barriers for them in conducting larger research studies.

## Novel trial designs

The standard trial designs detailed in epidemiology textbooks are on the path of becoming obsolete. **Dr Nadia Harbeck (Germany)**, the current ESMO Director of Education and Subject Editor Breast Cancer for the ESMO Guidelines, accompanied by **Dr Christina Yap (UK)** and **Dr Benjamin Haibe-Kains (Canada)** talked about adaptive trial designs and novel trial designs such as basket trial, platform trial, and umbrella trial. In basket trial, the effect of a drug on a mutation is checked on different tumour types. In platform trial, under a single overarching protocol, multiple treatments are evaluated. In umbrella trial, the effects of different drugs on various types of mutations within a single cancer type are evaluated. Researchers are encouraged to develop novel trial designs to improve efficiency, reduce costs, and minimise adverse effects of drugs.

## Pan-Asian Guidelines Adaptation (PAGA)

Considering many clinical guidelines are from the studies conducted in high-income countries in the western part of the world, young oncologists in Asia face challenges while providing treatment. The ESMO Pan-Asian Guidelines Adaptation (PAGA) project was started in 2017 to address this issue and is currently supported by ten cancer societies from various Asian countries. At ESMO Asia Congress 2024, three PAGA guidelines were adopted for epithelial ovarian cancer, oncogene-addicted metastatic non-small cell lung cancer, and biliary tract cancers. Although the impact of the PAGA guidelines might be known after 5–10 years, this is a significant step for improving cancer treatment in Asia. The development of PAGA guidelines for pancreatic cancer is currently in progress and is expected be adapted during the upcoming ESMO Asia Congress 2025 (December 5–7).

*The Lancet Regional Health—Southeast Asia* believes publication of this News article would encourage manuscript submissions from researchers and clinicians working on improving cancer care in Asia.

